# Ultrasound Screening in the First and Second Trimester of Pregnancy for the Detection of Fetal Cardiac Anomalies in a Low-Risk Population

**DOI:** 10.3390/diagnostics15060769

**Published:** 2025-03-19

**Authors:** Aura Iuliana Popa, Nicolae Cernea, Marius Cristian Marinaș, Maria Cristina Comănescu, Ovidiu Costinel Sîrbu, Dragoș George Popa, Larisa Pătru, Vlad Pădureanu, Ciprian Laurențiu Pătru

**Affiliations:** 1 Doctoral School, University of Medicine and Pharmacy of Craiova, 200349 Craiova, Romania; aura.nedianu@yahoo.com; 2 Department of Obstetrics and Gynecology, University of Medicine and Pharmacy of Craiova, 200349 Craiova, Romania; nicolae.cernea@gmail.com (N.C.); ovidiusoc@gmail.com (O.C.S.); patru.ciprianl@gmail.com (C.L.P.); 3 Department of Human Anatomy, University of Medicine and Pharmacy of Craiova, 200349 Craiova, Romania; cristinacomanescu85@gmail.com; 4 Department of Plastic Surgery, University of Medicine and Pharmacy of Craiova, 200349 Craiova, Romania; 5 Department 9, University of Medicine and Pharmacy of Craiova, 200349 Craiova, Romania; larisa.patru@umfcv.ro; 6 Department of Internal Medicine, University of Medicine and Pharmacy of Craiova, 200349 Craiova, Romania; vlad.padureanu@umfcv.ro

**Keywords:** echocardiography, fetus, congenital heart disease, prenatal diagnosis

## Abstract

**Background/Objectives:** Congenital heart disease (CHD) is the most common birth defect, an important cause of morbidity and mortality, with a reported prevalence of 5–12 per 1000 live births. The aim of our study was to identify the role of fetal morphological ultrasound examination in the first and second trimester of pregnancy in the detection of fetal congenital cardiac anomalies in a low-risk population. **Methods:** We performed a retrospective study in a tertiary fetal medicine center in Emergency Hospital Craiova, Romania. The longitudinal analysis combined first- and second-trimester screening using improved ultrasound protocols. Our study evaluated 8944 pregnant women with singleton pregnancies in a 6-year period between January 2018 and December 2023. All ultrasound examinations were performed using a standard extended protocol according to the main guidelines’ recommendations for the detection of fetal anomalies. **Results:** In the first trimester of pregnancy, 37 cases with cardiac anomalies were diagnosed. Thirteen of these cases were associated with genetic anomalies (Down syndrome—eight cases, Edwards syndrome—four cases, Turner syndrome—one case). Some of these pregnancies were associated with at least one of the minor ultrasound markers (inverted ductus venosus, abnormal flow in the tricuspid valve, presence of choroid plexus cysts, absent/hypoplastic nasal bone). In the second trimester of pregnancy, 17 cases of cardiac anomalies were diagnosed. From these cases, one was associated with genetic anomalies (DiGeorge Syndrome), and one case developed hydrops and delivered prematurely in the early third trimester. **Conclusions**: Ultrasound screening for the detection of congenital heart disease is feasible early in pregnancy, but some anomalies would be obvious later in pregnancy. An early diagnosis using an extended ultrasound protocol, genetic testing, and a multidisciplinary evaluation would improve the prognosis and the overall survival rate by delivering in a tertiary center that allows for rapid cardiac surgery in dedicated cases.

## 1. Introduction

Congenital heart disease (CHD) is the most common birth defect, an important cause of morbidity and mortality, with a reported prevalence of 8 per 1000 live births [1]. The early diagnosis of severe cardiac anomalies reduces the psychological impact of the pregnant woman, offers time to make therapeutic decisions, and offers families the possibility of counseling on the evolution of the detected anomalies and neonatal complications [2]. It also offers the necessary time for performing prenatal genetic tests [3,4] and decreases the delay of pregnancy termination.

The use of standardized ultrasound protocols during the first trimester (11–13 weeks + 6 days) will increase the early detection rates of congenital heart anomalies [4,5]. Previous studies [3,4] have demonstrated that fetal cardiac anomalies can be divided into malformations that are always detectable in the first trimester, malformations that cannot be detected in the first trimester, and malformations that can only sometimes be detected in the first trimester. The importance of both abdominal and transvaginal scanning in the early detection of fetal cardiac anomalies has also been demonstrated not only among the high-risk population but also among the low-risk population [5,6].

Severe cardiac anomalies are associated with a poor prognosis after surgical repair; therefore, the early detection of these anomalies and the decision of pregnancy termination can be considered an appropriate management [7]. The early detection of severe anomalies reduces the physical and mental stress of the pregnant woman and offers the possibility of pregnancy termination within the legal term [8]. There are several authors [7,8,9] who described the detection of CHD at a gestational age lower than 11 weeks, but others consider that we must be careful because of the psychological impacts on the parents due to the false positive rate at this gestational age.

Screening performances for CHD in the first trimester of pregnancy are influenced by the gestational age, sonographer experience, quality of equipment used, and time allowed for examination. There are also some benefits described in the evaluation of the fetal heart in the first trimester compared to the second one, mostly due to increased fetal movements and a lower ossification of the fetal bone structures.

Karim et al. [10], in a recent meta-analysis, showed that the sensitivity of ultrasound screening for CHD is increasing from 32% for the evaluation of only the four-chamber view, up to 56% for the assessments of the four-chamber view and outflow tracts, to more than 80% if the both structures are evaluated with gray-scale and color Doppler. Another important issue is the safety of using ultrasound in pregnancy, especially in the first trimester [11]. Previous prospective studies [12,13] showed that safety is possible if the threshold of the thermal index and mechanical index used is below 1.0 due to the technological progress of ultrasound equipment used.

The aim of our study was to identify the role of fetal morphological ultrasound examination in the first and second trimester of pregnancy in the detection of fetal congenital cardiac anomalies in a low-risk population and the role of the multidisciplinary evaluation of these cases.

## 2. Materials and Methods

The study population included women with no previous CHD, autoimmune diseases, hypertension, diabetes, or any other previous exposure to teratogens. This study was approved by the ethics committee of the University of Medicine and Pharmacy of Craiova. We performed a retrospective study in a tertiary fetal medicine center in the Emergency County Hospital from Craiova, Romania. Our study evaluated 8944 singleton pregnancies during a 6-year period between January 2018 and December 2023. All ultrasound examinations were performed using a standard extended protocol according to the main guidelines’ recommendations for the detection of pregnancy anomalies [14,15].

### 2.1. First Trimester Scanning and Sections

**Figure 1:**
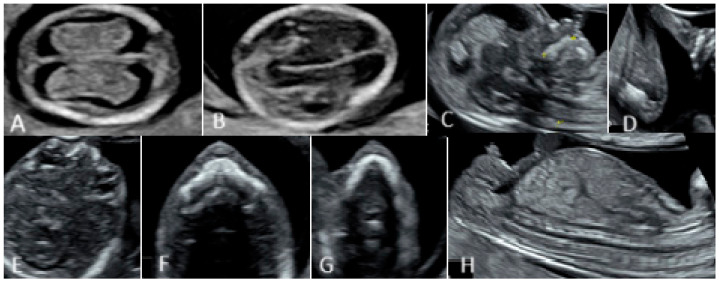
First-trimester morphological assessment of the fetus.(**A**,**B**) Head and brain. (**C**) Profile. (**D**) Limbs. (**E**–**G**) Fetal face (orbits, palate, mandibula). (**H**) Chest and abdomen.

The first-trimester evaluations were performed between 11 and 13 weeks and six days using ultrasound equipment with a high resolution, GE Voluson E8 (GE Healthcare, Zipf, Austria, Version 21.1), by the transabdominal and transvaginal approaches. The transvaginal method was used in cases of an unfavorable fetal position, maternal obesity, previous abdominal surgery, anterior position of the placenta, and uterine myomas/contractions. The used protocol involved standard sections for assessing the fetal head, abdominal wall, diaphragm, stomach, limbs, umbilical cord insertion, kidneys, bladder, and spine (Figure 1).

**Figure 2:**
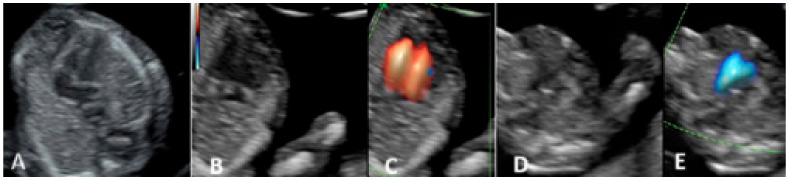
First-trimester heart protocol. (**A**) Four-chamber view. (**B**) Atrio-ventricular flows—Grey Scale. (**C**) Atrio-ventricular flows—Color Doppler Mode. (**D**) 3-vessel view—Grey Scale. (**E**) 3-vessel view—Color Doppler Mode.

The fetal heart protocol included the examination of the fetal heart in gray-scale and color doppler mode. The examination started with the evaluation of the heart situs, cardiac axis, and chest heart ratio (no more than one-third of the chest area) and continued with the four-chamber view (4CV) in gray-scale and the outflow tract views in color doppler mode. In the 4CV section, we evaluated the offsetting of the atrio-ventricular valves, right- and left-sided structures approximately equal, and the atrio-ventricular septum. The left and right outflow tracts were evaluated by size (equal in size), and the examination ended with the 3-vessel view (3VV) color Doppler mode demonstrating the confluence of the two arterial arches on the left side of the spine (Figure 2).

### 2.2. Second Trimester Scanning and Sections

**Figure 3:**
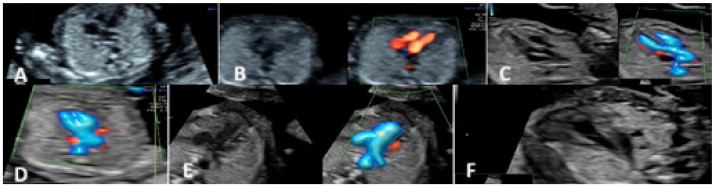
Second-trimester heart protocol. (**A**) Four-chamber view—Grey Scale. (**B**) Atrio-ventricular flows—Left side—Grey Scale and Right side—Color Doppler Mode. (**C**) Aorta—Left side in Grey Scale and Right side in Color Doppler Mode. (**D**) Crossing plane. (**E**) Pulmonary artery—Left side in Grey Scale and Right side in Color Doppler Mode. (**F**) 3-vessel and trachea view—Grey Scale.

The second-trimester ultrasound evaluation was performed between 20 and 24 weeks of gestation. The evaluation of the fetal heart started with the examination of the situs, cardiac axis, and chest heart ratio followed by the four-chamber view, outflow tract view (aorta and pulmonary artery), 3-vessel view (3VV), and 3-vessel and trachea view (3VT) and axial section for the evaluation of the interventricular septum and sagittal section (Figure 3).

### 2.3. Inclusion and Exclusion Criteria

The study inclusion criteria were singleton pregnancies that had a first-trimester dating scan between 7 and 10 weeks of gestation. The exclusion criteria were pregnancies over 14 weeks of gestation or pregnancies that ended by miscarriage or stillbirth without perinatal autopsy.

### 2.4. Statistical Analysis

Statistical analysis was performed using a database built in Microsoft Excel Version 2502 (Microsoft Corporation, Redmond, WA, USA) and IBM SPSS Statistics 26.0 (IBM Corporation, Armonk, NY, USA). Abnormal cases are reported in tables, produced by either MS Excel or SPSS. For continuous variables, we report average and standard deviation as well as medians for a rough assessment of asymmetry of distribution. The rates of anomalies and malformations are reported as percentages of different abnormalities out of the total number of abnormalities.

## 3. Results

### 3.1. First-Trimester Evaluation

**Figure 4:**
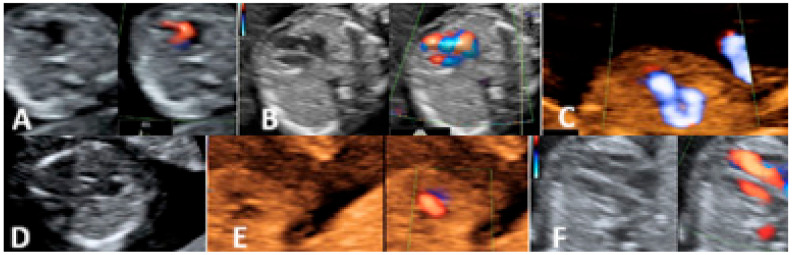
(**A**) Atrio-ventricular septal defect (Right side—Grey Scale and Left side—Color Doppler Mode). (**B**) Hipoplastic left heart ((Right side—Grey Scale and Left side—Color Doppler Mode) (**C**) Right aortic arch (Sepia-Color Doppler Mode). (**D**) Atrio-ventricular septal defect—Grey Scale) (**E**) Atrio-ventricular septal defect. (Right side—Sepia and Left side—Color Doppler Mode). (**F**) Hipoplastic left heart (Right side—Grey Scale and Left side—Color Doppler Mode).

In the first trimester of pregnancy, 37 cases with cardiac anomalies were diagnosed. Thirteen of these cases were associated with genetic anomalies (Down syndrome—eight cases, Edwards syndrome—four cases, Turner syndrome—one case). Some of these pregnancies were associated with at least one of the minor ultrasound markers (inverted ductus venosus, abnormal flow in the tricuspid valve, presence of choroid plexus cysts, absent/hypoplastic nasal bone) (Figure 4 and Table 1). 

The average age was 26.8 ± 5.6 years, and the median age was 17 years. The BMI ranged between 18.4 kg/m^2^ and 37 kg/m^2^, with an average of 23.9 kg/m^2^ ± 3.9 kg/m^2^ and a median of 22.7 kg/m^2^.

Increased nuchal translucency > 99th percentile was seen in 15 cases. Compared with normal fetuses, the incidence of NT thickening was significantly increased in fetuses with major cardiac anomalies (40.5%). Transabdominal ultrasound was performed in all of the cases. An additional transvaginal ultrasound was performed when the transabdominal was considered inadequate due to poor visualization in 1234 cases. The average time taken for a transabdominal examination only for the cardiac scan was 23 min. The minimum duration was 8 min, and maximum duration was 38 min. The mean duration for the transvaginal and transabdominal examinations was 32.4 min. A total of 45 cases had refusal of the transvaginal ultrasound, and in these cases, we recalled the patients for another transabdominal ultrasound at a 10–14 day interval. Most of the fetal cardiac examinations were performed in a single sitting. Two sittings were needed in 45 of the cases, and three sittings were needed in two cases.

In the first trimester, we found an additional minor marker associated in a total of 23 patients, four cases with inverted ductus venosus, six cases diagnosed with choroid plexus cyst, five cases of intracardiac focus, two cases with absent nasal bone, and four cases with tricuspid regurgitation. From the four cases diagnosed with tricuspid regurgitation, in two cases, we also detected a structural cardiac anomaly. All the cases associated with cardiac and genetic anomalies had a termination of the pregnancy. In these cases, the genetic diagnosis was made by chorionic villous sampling and amniocentesis.

In the first trimester, we diagnosed nine cases of Tetralogy of Fallot/common arterial trunk, seven cases of hypoplastic left heart/coarctation of the aorta, five cases of right aortic arch, four cases of atrio-ventricular septal defects, double outlet right ventricle in three cases, D-transposition of the great arteries in three cases, univentricular heart in two cases, cardiac complex malformation in two cases, tricuspid valve atresia with VSD in one case, and major VSD in one case (Table 2).

### 3.2. Second-Trimester Evaluation

**Figure 5:**
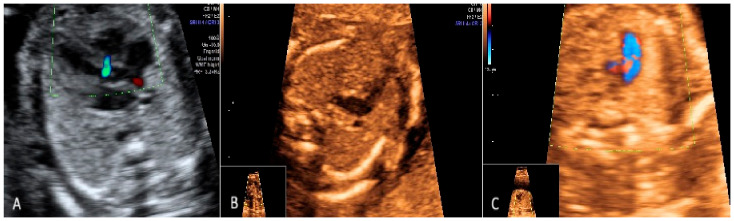
(**A**) Atrio-ventricular septal defect (Grey scale). (**B**) Transposition of the great arteries (Sepia). (**C**) Coarctation of aorta (Sepia -Color Doppler Mode).

In the second trimester of pregnancy, 17 cases of cardiac anomalies were diagnosed (Figure 5). We detected three cases of Tetralogy of Fallot, five cases of VSD, fetal paroxysmal supraventricular tachycardia in two cases, coarctation of the aorta in two cases, and rhabdomyoma in one case. From these cases, one was associated with genetic anomalies (DiGeorge Syndrome), two cases developed hydrops and delivered prematurely in the early third trimester, and in one case, spontaneous abortion occurred at 23 weeks due to premature rupture of membranes (Table 2).

The case diagnosed with the cardiac anomaly due to aneurysmal of the vein of Galen was associated with cardiac failure and intrauterine fetal death at a gestational age of 28 weeks.

In this case, a fetal postnatal autopsy was performed, which confirmed the structural anomaly. After catheterization of the right and persistent left superior vena cava and aorta, a blue-colored gelatine was injected into the venous system, and a green-colored gelatine was injected into the aorta. We observed a very good passage in the venous system and high resistance pressure in the arterial system due to massive thrombosis. We identified a dilated vein of Galen and straight sinus by the blue-colored gelatine.

One case diagnosed with paroxysmal supraventricular tachycardia was converted to sinusal rhythm. In this case, at the moment of diagnosis, there was no fetal hydrops detected and a delivery at 37 weeks of gestation with good prognosis and outcome.

The other case detected with paroxysmal supraventricular tachycardia at 31 weeks of gestation presented fetal massive hydrops. After 3 days with transitory short episodes of sinusal conversion, a preterm rupture of the amniotic membranes occurred and preterm labor. The neonatal prognosis was poor, and the fetus died after 36 h.

The rest of the cases diagnosed in the second trimester were referred to regional cardiac surgery centers. One of the cases diagnosed with severe coarctation of the aorta had severe intrauterine growth restriction and a preterm delivery at 33 weeks of gestation. Due to the neonatal prematurity complications and restriction, the fetus died at 72 h after delivery. The remaining two cases diagnosed with coarctation of the aorta, the three cases of Tetralogy of Fallot, and the five cases diagnosed with VSD underwent postpartum cardiac surgery, and no complications were reported due to the surgical intervention. The neonatal follow-up was performed by a pediatric cardiologist until 1.5 years after birth with satisfactory clinical evolution.

In the case diagnosed with L-transposition of the great arteries, after the couple was counseled, they opted for termination of the pregnancy.

The discordance between the detection rates in the first and second trimester was due to the high detection rate in the first trimester.

A z-test for comparing proportions showed that there was a statistically significant difference between the proportion of subjects diagnosed with cardiac anomalies in the first trimester and those diagnosed in the second trimester of pregnancy (z = 2.71, *p* < 0.01).

**Figure 6:**
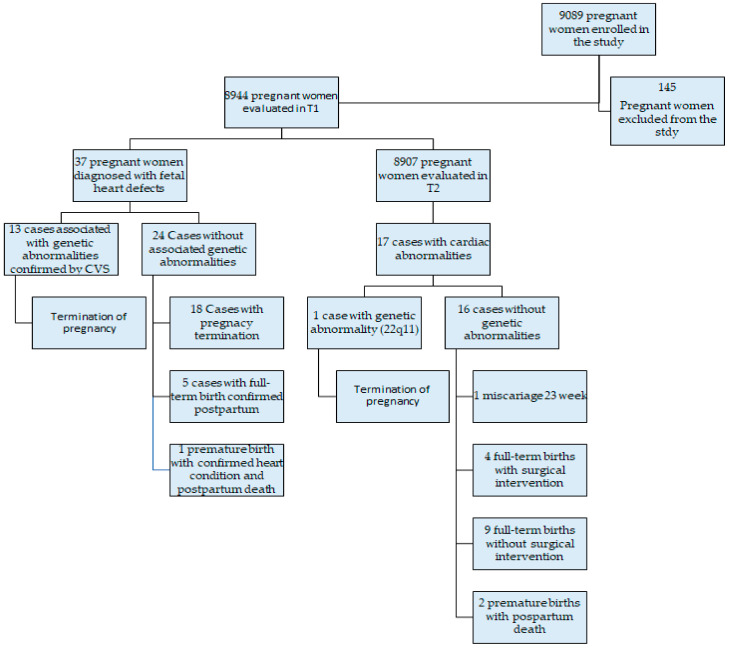
The diagnosis algorithm for the study population.

The pregnant women enrolled in the study were evaluated according to the diagnosis algorithm (Figure 6).

## 4. Discussion

Ultrasound screening for congenital heart disease in the first trimester of pregnancy may sometimes be a real challenge for the sonographer because of the size of the fetus and additional factors like maternal obesity, fetal position, uterine myomas/contractions, anterior position of the placenta, and previous surgical intervention of the mother [4,5].

Factors affecting the ability to evaluate fetal heart anomalies during a scan at 11–13 weeks are dependent on the operator’s skills and equipment. Operators should have a good scanning technique and a high level of expertise in fetal echocardiography. For a better visualization, an image optimization should be performed. Because, in the first trimester, the fetal heart dimensions are only between 6 and 10 mm, the ultrasound machine used for the assessment of the fetal heart should include a wide spectrum of modern image enhancers [16].

After 11 weeks of gestation, a standard evaluation of the four-chamber view can be performed in gray-scale (98% of cases) and Doppler color (86% of cases) [16,17]. Better screening performance was reported after 13 weeks of gestation because of the morphological changes in the fetal heart [18]. Some cardiac abnormalities, such as cardiac tumors, aortic and pulmonary stenosis, and cardio myopathies, are not evident until later in pregnancy and can progress into more severe malformations. These cardiac abnormalities often go undetected, even at the routine anomaly scan in the second trimester [18].

An early diagnosis of fetal congenital anomalies presents multiple benefits [19]. One of them is that some of the severe CHDs have a poor prognosis, and another is that patients might have an option for an early pregnancy termination [20]. Optimization of high-quality images plays an important role in the first trimester; an adequate insonation angle, doppler settings-gain, pulse repetition frequency (PRF), and wall motion filter (WMF) are very important. Usually, it is recommended to alternate low PRF/WMF and high PRF/WMF for a better visualization of the morphology of the ventricles/heart and great vessels [21]. The cardiac axis is modified in most cases diagnosed with severe CHDs (in over 90% of cases), mostly as a left deviation [22].

Abnormal DV flow was reported in the first and second trimester of pregnancy to be associated with CHD, tachycardia, cardiomyopathy, and increased right ventricular overload. In such cases, the atrial contraction is against the increased impedance to forward flow, resulting in a reversed flow in the DV that leads to a negative a-wave.

If an inverted DV flow is detected in the first trimester, one should pay attention to cardiac evolving diseases in the second trimester. Also, this may lead to fetal hydrops. Our study results show that this minor anomaly can also be associated with other structural and genetic anomalies [23].

When tricuspid regurgitation is observed, the risk of CHDs is increased by eight-fold. A tricuspid regurgitation was detected in higher rates in fetuses with aneuploidy [24,25]. A Doppler assessment of the ductus venosus should be included routinely in the first trimester ultrasound examination because, when an abnormal flow is present, a major CHD is seen in 1/3 of the cases [26].

Genetic abnormalities are observed in up to one-third of prenatally diagnosed CHDs [27]; the prevalence of CHDs increased with the increasing of the NT thickness in a stronger association with hypoplastic left heart syndrome and coarctation of the aorta [28]. Our study results show similar rates. The mechanism of an increased NT in fetuses with CHD is not fully understood. A proposed theory includes narrowing of the aortic isthmus accompanied by narrowing of the aortic valve and ascending aorta, leading to diversion of more blood to the head and neck [29].

Previous studies reported first-trimester detection rates over 90% for hypoplastic hearth syndrome, atrio-ventricular septal defects (AVSD), and tricuspid or pulmonary atresia [3,10]. On the other hand, the diagnosis of small ventricular septal defects or conotruncal anomalies is a real challenge at this stage of gestation. In some cases diagnosed with CHDs, prognosis indicators in the first trimester are difficult to appreciate, for example, diminution of the pulmonary artery diameter in cases diagnosed with Tetralogy of Fallot (this progressive narrowing of the pulmonary artery is seen in 25% of cases) [30]. Some CHDs (aortic stenosis [31], pulmonary stenosis [32], Ebstein anomaly [33]) are known to evolve in the second and third trimester of pregnancy and can be missed at the time of the routine second-trimester ultrasound screening. A discrete coarctation of the aorta can be misdiagnosed due to the patency of the ductus arteriosus, and also, secundum atrial defects can be misdiagnosed due to the patency of the foramen ovale.

Our study confirms the diagnostic role of ultrasound starting from the late first trimester. Our data support the use of a standard morphological protocol when performing the examination. Using this protocol, we succeeded in detecting many major and minor structural anomalies, especially in the first trimester. The detection rates, as our study results show, are similar to the data obtained from the current literature [2,20]. Despite all this progress, a publication of the Society of Thoracic Surgeons Congenital Heart Surgery [34] describes only a 57% detection rate of CHD in the case of an abnormality of the four-chamber view, and only 32% detection rates in the case of an abnormality of the great vessels in contrast with CNS anomalies, who reported better detection rates for the first trimester [35].

Ultrasound training is very important in the case of performing ultrasound screening for the detection of CHD [36]; an early detailed assessment of the fetal heart requires a high level of expertise in fetal echocardiography.

The limitations of this study are the following: a low number of detected cases, due to the study design; a low-risk population, and a single-center study.

The strengths of this study are the following: important contributions, such as the longitudinal analysis of combined first- and second-trimester screening, and the detailed ultrasound protocols.

## 5. Conclusions

Ultrasound screening for the detection of congenital heart disease is feasible early in pregnancy, but some anomalies would be obvious later in pregnancy. An early diagnosis will improve the prognosis and overall survival rates because of the performance of genetic tests; evaluation in a multidisciplinary team, formed by a fetal medicine specialist, geneticist, pediatric cardiologist, and cardiac surgeons; and also the delivery in a tertiary center, which allows for rapid cardiac surgery in dedicated cases. With the increasing quality of imaging and the use of artificial intelligence (AI), in the future, we hope to improve the diagnosis rates and to eliminate discrepancies between the tertiary centers and basic centers in diagnostic rates.

Although some fetal anomalies cannot be diagnosed in the first trimester, the major structural anomalies can. In relation to this, major congenital heart anomalies can be diagnosed in the late first trimester by using the obstetrical ultrasound guidelines’ recommendations.

The detection of major CHDs at 11–13 weeks of gestation is improved by the association with other structural and genetic ultrasound markers. The use of both transabdominal and transvaginal ultrasound improves the anomaly detection rate. However, the limitations of fetal cardiac assessment in the first trimester and the evolving heart diseases during pregnancy should motivate the practitioners to continue the cardiac evaluation in the second and third trimester of pregnancy. Our study results demonstrated that, although we can detect most of the severe cardiac anomalies in the first trimester, this primary evaluation should not replace further evaluation.

## Figures and Tables

**Table 1:** First-trimester association between cases diagnosed with chromosomal ultrasound markers and structural anomalies.

Anomaly	Number of Cases			Percent
Nuchal translucency (NT)	NT < 3.5 mm—22	NT > 3.5 mm—15		15/37 (40.5%)
Ductus venosus (DV)	Normal DV—33	Inverted DV—4		4/37 (10.8%)
Choroid plexus cyst (CPC)	Absent—31	Present—6		6/37 (16.2%)
Intracardiac focus (FI)	Absent—32	Present—5		5/37 (13.5%)
Hypoplastic or absent nasal bone (NB)	Present—31	Hypoplastic—4	Absent—2	4/37 (10.8%) and 2/37 (5.4%)
Tricuspid regurgitation (RT)	Absent—32	Present—4	Absence of flow in the tricuspid valve—1	4/37 (10.8%) and 1/37 (2.7%)

**Table 2:** First- and second-trimester distribution of the cases detected with anomalies.

Cardiac Abnormalities	Cases		Percentage	
	First Trimester	Second Trimester	First Trimester	Second Trimester
Common arterial trunk/Tetralogy of Fallot	9		9/37 (24.3%)	
Hypoplasia of the left heart/coarctation of the aorta	7		7/37 (18.9%)	
Right aortic arch	5	1	5/37 (13.5%)	1/17 (5.88%)
Atrio-ventricular septal defects	4		4/37 (10.81%)	
Double outlet right ventricle	3		3/37 (8.11%)	
D-transposition of the great arteries	3		3/37 (8.11%)	
Univentricular heart	2		2/37 (5.41%)	
Complex cardiac malformation	2		2/37 (5.41%)	
Tricuspid valve atresia with VSD	1		1/37 (2.7%)	
Major interventricular septal defect (VSD)	1		1/37 (2.7%)	
Tetralogy of Fallot		3		3/17 (17.65%)
Membranous VSD		5		5/17 (29.41%)
Supraventricular tachycardia (SVT)		2		2/17 (11.76%)
Coarctation of the aorta		3		3/17 (17.65%)
L-transposition of large vessels		1		1/17 (5.88%)
Cardiomegaly due to aneurysm of the vein of Galen		1		1/17 (5.88%)
Rhabdomyoma		1		1/17 (5.88%)
TOTAL Total	37	17	100%	100%

## Data Availability

All data presented here are available from the authors upon reasonable request.

## References

[1] Bouma B.J., Mulder B.J. (2017). Changing Landscape of Congenital Heart Disease. Circ. Res..

[2] Bakker M.K., Bergman J.E.H., Krikov S., Amar E., Cocchi G., Cragan J., Walle H.E.K.d., Gatt M., Groisman B., Liu S. (2019). Prenatal diagnosis and prevalence of critical congenital heart defects: An international retrospective cohort study. BMJ.

[3] Syngelaki A., Hammami A., Bower S., Zidere V., Akolekar R., Nicolaides K.H. (2019). Diagnosis of fetal non-chromosomal abnormalities on routine ultrasound examination at 11–13 weeks’ gestation. Ultrasound Obstet. Gynecol..

[4] Syngelaki A., Chelemen T., Dagklis T., Allan L., Nicolaides K.H. (2011). Challenges in the diagnosis of fetal non-chromosomal abnormalitiesat 11–13 weeks. Prenat. Diagn..

[5] Ye B., Wu Y., Chen J., Yang Y., Niu J., Wang H., Wang Y., Cheng W. (2021). The diagnostic value of the early extended fetal heart examination at 13 to 14 weeks gestational age in a high-risk population. Transl. Pediatr..

[6] Hernandez-Andrade E., Patwardhan M., Cruz-Lemini M., Luewan S. (2017). Early evaluation of the fetal heart. Fetal Diagn. Ther..

[7] Sun H.Y. (2021). Prenatal diagnosis of congenital heart defects: Echocardiography. Transl. Pediatr..

[8] Kashyap N., Pradhan M., Singh N., Yadav S. (2015). Early detection of fetal malformation, a long distance yet to cover! present status and potential of first trimester ultrasonography in detection of fetal congenital malformation in a developing country: Experience at a tertiary care centre in India. J. Pregnancy.

[9] Rolnik D.L., Wertaschnigg D., Benoit B., Meagher S. (2020). Sonographicdetection of fetal abnormalities before 11 weeks of gestation. Ultrasound Obstet. Gynecol..

[10] Karim J.N., Bradburn E., Roberts N., Papageorghiou A.T. (2022). First-trimester ultrasounddetection of fetal heart anomalies: Systematic review and metaanalysis. Ultrasound Obstet. Gynecol..

[11] Torloni M.R., Vedmedovska N., Merialdi M., Betrán A.P., Allen T., González R., Platt L.D. (2009). Safety of ultrasonography in pregnancy: WHO systematic review of the literature and meta-analysis. Ultrasound Obstet. Gynecol..

[12] Nemescu D., Berescu A., Rotariu C. (2015). Variation of safety indices during in the learning curve for color Doppler assessment of the fetal heart at 11+0 to 13+6 weeks’ gestation. Med. Ultrason..

[13] Sinkovskaya E., Dall’Asta A., Maršál K., Lees C., Board of the International Society of Ultrasound in Obstetrics and Gynecology (ISUOG) (2021). ISUOG statement on the safe use of Doppler for fetal ultrasound examination in the first 13 + 6 weeks of pregnancy (updated). Ultrasound. Obstet. Gynecol..

[14] Carvalho J.S., Allan L.D., Chaoui R., Copel J.A., DeVore G.R., Hecher K., Lee W., Munoz H., Paladini D., Tutschek B. (2013). ISUOG Practice Guidelines (updated): Sonographic screening examination of the fetal heart. Ultrasound Obstet. Gynecol..

[15] Bromley B., Henningsen C., Jones D.C., Timor-Tritsch I., Simpson L.L., Thiel L. (2021). AIUM Practice Parameter for the Performance of Detailed Diagnostic Obstetric Ultrasound Examinations Between 12Weeks 0Days and 13 Weeks 6 Days. J. Ultrasound Med..

[16] Hutchinson D., McBrien A., Howley L., Yamamoto Y., Sekar P., Motan T., Jain V., Savard W., Hornberger L.K. (2017). First-trimester fetal echocardiography: Identification of cardiac structures for screening from 6 to 13 weeks’ gestational age. J. Am. Soc. Echocardiogr..

[17] Quarello E., Lafouge A., Fries N., Salomon L.J. (2017). Basic heart examination: Feasibility study of first-trimester systematic simplified fetal echocardiography. Ultrasound Obstet. Gynecol..

[18] Bilardo C.M., Chaoui R., Hyett J.A., Kagan K.O., Karim J.N., Papageorghiou A.T., Poon L.C., Salomon L.J., Syngelaki A., Nicolaides K.H. (2023). ISUOG practice guidelines(updated): Performance of 11–14-week ultrasound scan. Ultrasound Obstet. Gynecol..

[19] Hui L., Johnson J., Norton M.E. (2022). ISPD 2022 debate—When offering a first trimester ultrasound at 11 + 0 to 13 + 6 weeks, a detailed review of fetal anatomy should be included. Prenat Diagn..

[20] Iliescu D., Tudorache S., Comanescu A., Antsaklis P., Cotarcea S., Novac L., Cernea N., Antsaklis A. (2013). Improved detection rate of structural abnormalities in the first trimester using an extended examination protocol: Early anomaly scan. Ultrasound Obstet. Gynecol..

[21] Herghelegiu C.G., Panaitescu A.M., Duta S., Vayna A.M., Ciobanu A.M., Bulescu C., Ioan R.G., Neacsu A., Gica N., Veduta A. (2021). Ultrasound Patterns in the First Trimester Diagnosis of Congenital Heart Disease. J. Clin. Med..

[22] Jung Y.J., Lee B.R., Kim G.J. (2020). Efficacy of fetal cardiac axis evaluation in the first trimester as a screening tool for congenital heart defect or aneuploidy. Obstet. Gynecol. Sci..

[23] Iliescu D.G., Nagy R.D., Carbunaru O., Comanescu A., Ruican D., Burada F., Ioana M., Streata I., Stoica A., Gheonea M. (2019). EP15.20: Agenesis of ductus venosus: Predictors and new anatomical variants. Ultrasound Obstet. Gynecol..

[24] Huggon I.C. (2003). Tricuspid regurgitation in the diagnosis of chromosomal anomalies in the fetus at 11–14 weeks of gestation. Heart.

[25] Faiola S., Tsoi E., Huggon I.C., Allan L.D., Nicolaides K.H. (2005). Likelihood ratio for trisomy 21 in fetuses with tricuspid regurgitation at the 11 to 13+6-week scan: Trisomy 21 in fetuses with tricuspid regurgitation. Ultrasound Obstet. Gynecol..

[26] Timmerman E., Clur S.A., Pajkrt E., Bilardo C.M. (2010). First-trimester measurement of the ductus venosus pulsatility index and the prediction of congenital heart defects. Ultrasound Obstet. Gynecol..

[27] Carvalho J.S. (2005). The fetal heart or the lymphatic system or…? the quest for the etiology of increased nuchal translucency. Ultrasound Obstet. Gynecol..

[28] Williams K., Carson J., Lo C. (2019). Genetics of congenital heart disease. Biomolecules.

[29] Hyett J., Perdu M., Sharland G., Snijders R., Nicolaides K.H. (1999). Using fetal nuchal translucency to screen for major congenital cardiac defects at 10e14 weeks of gestation: Population based cohort study. BMJ.

[30] De Robertis V., Persico N., Volpe G., Rembouskos G., Fabietti I., Olivieri C., Giudicepietro A., Volpe P. (2020). Tetralogy of Fallot and outlet ventricular septal defect with anterior malalignment detected at early fetal echocardiography. Fetal Diagn. Ther..

[31] Freud L.R., Moon-Grady A., Escobar-Diaz M.C., Gotteiner N.L., Young L.T., McElhinney D.B., Tworetzky W. (2015). Low rate of prenatal diagnosis among neonates with critical aortic stenosis: Insight into the natural history in utero. Ultrasound Obstet. Gynecol..

[32] Ronai C., Freud L.R., Brown D.W., Tworetzky W. (2020). Low. prenatal detection rate of valvar pulmonary stenosis: What are we missing?. Prenat. Diagn..

[33] Tierney E.S.S., McElhinney D.B., Freud L.R., Tworetzky W., Cuneo B.F., Escobar-Diaz M.C., Ikemba C., Kalish B.T., Komarlu R., Levasseur S.M. (2017). Assessment of progressive pathophysiology after early prenatal diagnosis of the Ebstein anomaly or tricuspid valve dysplasia. Am. J. Cardiol..

[34] Quartermain M.D., Pasquali S.K., Hill K.D., Goldberg D.J., Huhta J.C., Jacobs J.P., Jacobs M.L., Kim S., Ungerleider R.M. (2015). Variation in prenatal diagnosis of congenital heart disease in infants. Pediatrics.

[35] Ungureanu D.R., Drăgușin R.C., Căpitănescu R.G., Zorilă L., Ofițeru A.M.I., Marinaș C., Pătru C.L., Comănescu A.C., Comănescu M.C., Sîrbu O.C. (2023). First Trimester Ultrasound Detection of Fetal Central Nervous System Anomalies. Brain Sci..

[36] Nemescu D., Onofriescu M. (2015). Factors affecting the feasibility of routine first-trimester fetal echocardiography. J. Ultrasound Med..

